# Ascites in the Course of Plasma Cell Myeloma Complicated by AL Amyloidosis

**DOI:** 10.4274/tjh.2016.0262

**Published:** 2018-03-06

**Authors:** Jakub Debski, Lidia Usnarska-Zubkiewicz, Katarzyna Kapelko-Słowik, Aleksander Pawluś, Urszula Zaleska-Dorobisz, Kazimierz Kuliczkowski

**Affiliations:** 1 Uniwersytet Medyczny im Piastow Slaskich we Wroclawiu, Department of Hematology, Blood Neoplasms and Bone Marrow Transplantation, Wroclaw, Poland; 2 Uniwersytet Medyczny im Piastow Slaskich we Wroclawiu, Department of Radiology, Wroclaw, Poland

**Keywords:** Myeloma, Amyloidosis, Ascites

A 60-year-old Caucasian male with plasma cell myeloma (PCM) immunoglobulin G (IgG) kappa, International Staging System stage 3, diagnosed 5 months ago, was admitted to the department of hematology due to progression of the disease. He had completed three cycles of chemotherapy comprising bortezomib, thalidomide, and dexamethasone; one cycle comprising vincristine, doxorubicin, and dexamethasone; and two cycles comprising lenalidomide and dexamethasone, without any clinically significant response. Three weeks before visiting the hospital, the patient also started complaining of progressive weakness, impaired respiratory function, and abdominal distension; an abdominal ultrasound at the time revealed hepatosplenomegaly with ascites, most likely associated with portal hypertension and protein disturbance, which initially he tolerated very well. Physical examination revealed crackles over the basal areas of the lungs, an enlarged spleen and liver, ascites (stage 2), and peripheral pitting edema. Bone marrow aspiration revealed that plasmacytes accounted for 58% of all nucleated cells. Laboratory tests revealed the following: serum monoclonal IgG, 88.4 g/L (normal: 8-17) and b2-microglobulin, 26.8 mg/L (normal: 1.09-2.53). An abdominal wall fat pad biopsy was positive for amyloid by Congo red staining; this correlated with elevated B-type natriuretic peptide levels (818.7 pg/mL; normal: 0-125). Peritoneal paracentesis was performed and 650 mL of red fluid was aspirated. Laboratory tests revealed a serum-ascites albumin gradient of 1.1 g/dL, with elevated lactate dehydrogenase. Microscopic examination of slide preparations revealed extensive monotonous infiltration by plasmacytes and plasmablasts with highly atypical nuclei and wide polymorphism; monoclonality (CD38+ CD56+ CD45+ CD138+ cyk+) was confirmed by immunophenotyping (Figure 1A). Computed tomography of the abdomen and thorax revealed interstitial changes in the lower lobes of the lungs; pathological contrast enhancement of enlarged (up to 16-20 mm in diameter) paraaortic, paratracheal, and mediastinal lymph nodes; hepatosplenomegaly with ascites and dilatation of the portal venous system; multiple infiltrations of the abdominal wall (described as peritoneal carcinomatosis); focal osteolysis of the thoracic and lumbar vertebrae; and enlargement of the right ventricle (Figures 1B and 1C). This clinical presentation reflected aggressive features of advanced, chemoresistant PCM with coexisting AL amyloidosis. Due to the high level of monoclonal proteins in the serum, we performed plasmapheresis and implemented a salvage chemotherapy regimen based on bendamustine. However, despite intensive treatment, the patient died of disease progression.

Ascites is an extremely rare extramedullary manifestation of a heterogeneous clinical entity such as PCM, although it is worth noting that it has a greater predilection for the IgA subtype than for IgG [1,2]. Similarly, as in the current case, the condition may have multifactorial etiology associated with PCM progression, i.e. infiltration of the liver, heart failure, renal failure, portal hypertension, amyloidosis, and, finally, peritoneal myelomatous deposits [3]. Despite multimodal treatment, including radiation therapy, plasmapheresis, systemic chemotherapy based on novel drugs, and hematopoietic stem cell transplantation, the appearance of ascites heralds a dismal prognosis; median overall survival is usually no longer than 2 months [2,4].

## Figures and Tables

**Figure 1:**
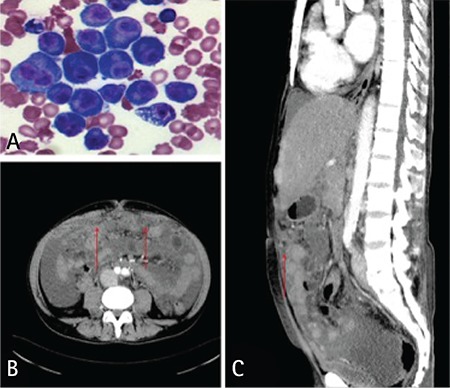
A) Microscopic evaluation of plasmacytes and plasmablasts in an ascitic fluid smear (modified Wright-Giemsa stain, 400x). B) Multiple myelomatous infiltrations of the peritoneal cavity (computed tomography scan, axial plane). C) Multiple myelomatous infiltrations of the peritoneal cavity (computed tomography scan, sagittal plane).

## References

[1] Morgan D, Cieplinski W (1985). Myelomatous ascites. Am J Med Sci.

[2] Mitra S, Mukherjee S, Chakraborty H, Bhattacharyya M (2015). IgG lambda myeloma presenting as plasmacytic ascites: case report and review of literature. Indian J Hematol Blood Transfus.

[3] Karp SJ, Shareef D (1987). Ascites as a presenting feature multiple myeloma. J R Soc Med.

[4] Kyle RA, Gertz MA, Witzig TE, Lust JA, Lacy MQ, Dispenzieri A, Fonseca R, Rajkumar SV, Offord JR, Larson DR, Plevak ME, Therneau TM, Greipp PR (2003). Review of 1027 patients with newly diagnosed multiple myeloma. Mayo Clin Proc.

